# GPR142 Agonists Stimulate Glucose-Dependent Insulin Secretion via Gq-Dependent Signaling

**DOI:** 10.1371/journal.pone.0154452

**Published:** 2016-04-22

**Authors:** Jingru Wang, Juan J. Carrillo, Hua V. Lin

**Affiliations:** 1 Lilly China Research and Development Center (LCRDC), Eli Lilly & Company, Shanghai, China; 2 Lilly Research Laboratories, Lilly Corporate Center (LCC), Eli Lilly & Company, Indianapolis, IN, United States of America; NIDCR/NIH, UNITED STATES

## Abstract

GPR142 is an islet-enriched G protein-coupled receptor that has been investigated as a novel therapeutic target for the treatment of type 2 diabetes by virtue of its insulin secretagogue activity. However, the signaling pathways downstream of GPR142 and whether its stimulation of insulin release is glucose-dependent remain poorly characterized. In this study, we show that both native and synthetic GPR142 agonists can activate Gq as well as Gi signaling when GPR142 is recombinantly expressed in HEK293 cells. However, in primary pancreatic islets, a native cellular system, the insulin secretagogue activity of GPR142 agonists only requires Gq activation. In addition, our results show that stimulation of insulin secretion by GPR142 in pancreatic islets is strictly glucose-dependent.

## Introduction

G protein-coupled receptors (GPCRs) are important cell surface mediators of signal transduction and a class of druggable targets that have been extensively studied for therapeutic intervention. GPR142 is a GPCR selectively activated by amino acids L-Tryptophan (L-Trp) and L-Phenylalanine with expression highly enriched in pancreatic islets in mouse and man [1](Lin et al., submitted) and was proposed to couple through Gq/11 signaling [2]. L-Trp and synthetic GPR142 agonists were reported to stimulate insulin secretion and improve glucose tolerance [3], suggesting an important role of this receptor in the regulation of glucose homeostasis. However, in the only report examining GPR142 mediated Gq signaling published to date [2], the receptor transcript (AB196529) was cloned from fetal brain and has a different predicted open reading frame from the transcript cloned from human pancreatic islets (XM_005257305.2)(Lin et al., submitted). Therefore, whether the GPR142 isoform expressed by islet cells couples through Gq bears reexamination. Furthermore, detailed studies of biological activities of this receptor, including potential signaling through additional Gα subunits and the contributions of specific signaling pathways responsible for GPR142 agonists’ insulin secretagogue activities, have not been reported.

Insulin secretagogues such as sulfonylureas and glinides have been widely used to treat patients with type 2 diabetes, but can increase the risk of hypoglycemia since they trigger insulin release from pancreatic islets independent of ambient glucose levels [4]. Glucose-dependent insulin secretagogue mechanisms, such as DPP-4 inhibitors and GLP-1 analogs [5], have emerged as preferred treatment alternatives due to their reduced hypoglycemic risk. As a novel insulin secretagogue mechanism, it is important to determine whether GPR142 agonists stimulate insulin secretion only in the presence of high ambient glucose, which is essential for a desired safety profile for the treatment of type 2 diabetes.

In this study, we performed a comprehensive survey of GPCR signaling pathways in a cell-based system using both L-Trp and a synthetic GPR142 agonist. We found GPR142 agonists activate both Gq and Gi-coupled signaling. While both Gq and Gi signaling contribute to GPR142-stimulated phosphorylation of extracellular signal-regulated kinase (ERK), only Gq is required for the dynamic mass redistributuion (DMR) signal observed upon GPR142 activation. Functional studies in pancreatic islets reveal that stimulation of insulin secretion by GPR142 activation is mediated through Gq-coupled signaling and strictly glucose-dependent. Together, our data indicate GPR142 agonism represents an attractive approach for the treatment of type 2 diabetes.

## Materials and Methods

### Cell lines and G protein inhibitors

HEK293 cells obtained from ATCC were used to generate HEK293-human GPR142 and HEK293-mouse Gpr142 stable cell lines. Briefly, HEK293 cells were transfected with pcDNA3.1 plasmids containing hGPR142 (RefSeq XM_005257305.2) or mGpr142 (RefSeq XP_006533129) insert using Lipofectamine Reagent (Invitrogen), and clones were selected with Dulbecco’s modified Eagle’s medium (DMEM) (Sigma) supplemented with 10% fetal bovine serum (FBS), 1% Antibiotic-Antimycotic, and 800 μg/ml G418 (Gibco). Stable cell lines were maintained in DMEM supplemented with 10% FBS, 1% Antibiotic-Antimycotic, and 800 μg/ml G418 and grown at 37°C in a humidified atmosphere of 5% CO_2_/95% air. Pertussis Toxin (PTX, Tocris Bioscience) was used to pre-treat cells overnight at a concentration of 100 ng/ml to inhibit Gi signaling. UBO-QIC was ordered from Institute of Pharmaceutical Biology, University of Bonn, and used to pre-treat cells for 1 hour at a concentration of 0.3 μM to inhibit Gq/11 signaling. PTX and UBO-QIC at the tested concentrations did not affect cell viability as measured by LDH release or cellular ATP content.

### IP-1 accumulation assay

HEK293-hGPR142 or HEK293-mGpr142 cells were plated in 96-well plates at 15000 cells per well and allowed 18 hours for attachment. After addition of GPR142 agonist compounds at varying concentrations, cells are incubated for 1 hour. Intracellular IP-1 levels were measured using an IP-One HTRF assay kit (Cisbio, 62IPAPEC) according to manufacturer’s protocol.

### cAMP assay

HEK293-hGPR142 or HEK293-mGpr142 cells were plated in 96-half well plates at 5000 cells per well and allowed 18 hours for attachment. After addition of 5 μM forskolin and compounds at varying concentrations, cells were incubated for 1 hour at room temperature. cAMP levels were measured using Cyclic AMP cell-based assay kit (Cisbio, 62AM4PEC) according to manufacturer’s protocol.

### FLIPR assay

HEK293-hGPR142 or HEK293-mGpr142 cells were plated in 96-well plates at 50000 cells per well and allowed 24 hours for attachment. After addition of Fluo-5 dye (Calcium-5 Assay Kit, Molecular Probes), cells were incubated for 1 hour at room temperature. Dye was then removed and substituted with assay buffer (HBSS with Ca/Mg, 20mM HEPES, 0.1% BSA). Compounds were added to plates, and signal was quantified by FLIPR tetra system (Molecular Devices).

### Cell-based quantitative detection of ERK phosphorylation

HEK293-hGPR142 or HEK293-mGpr142 cells were plated in 96-well plates at 45,000 per well and cultured overnight. The next day, cells were starved 2 hours in DMEM with 0.1% BSA. Then compounds at varying concentrations were added and cells were incubated for 1 hour at 37°C. Phosphorylated ERK levels were measured using Phospho-ERK (Thr202/Tyr204) Cellular Assay Kit (Cisbio, 64ERKPEH) according to manufacturer’s protocol.

### Label-free DMR assay

HEK293-hGPR142 or HEK293-mGpr142 cells were plated in EnSpire-LFC 384-well fibronectin coated microplate at 20000 cells per well, and then incubated overnight at 37°C. Prior to the assay, the plates were washed with assay buffer HBSS (20mM HEPES, pH 7.4) using BioTek wash station, with 30 μl assay buffer remaining in each well. The plates were equilibrated for 2 hours, and then 10 μl compound solution was added to each well. Quantification of DMR signals in living cells was performed with Corning Epic label-free technology by an EnSpire Multimode Plate Reader (PerkinElmer) for 5 hours. The maximum value obtained during the 5hr time course for each concentration was used to fit dose response curve.

### Mouse pancreatic islet insulin secretion

6 to 8-week-old male C57BL6 mice were obtained from Shanghai Laboratory Animal Center and housed with 4–5 animals per cage in rooms with 22–25°C ambient temperature and 12hr light/12hr dark cycles. Animals were allowed ad libitum access to standard rodent chow and water. All animal procedures were approved by the HD Biosciences (Shanghai) and Covance Shanghai’s Institutional Animal Care and Use Committees. Animals were euthanized by CO_2_ inhalation followed by subsequent cervical dislocation. Pancreatic islets were isolated by injection of collagenase XI solution (0.5 mg/mL, Sigma-Aldrich) into the common bile duct, excision of the pancreas, digestion at 37°C for 17 minutes, and Dextran density gradient separation. Isolated islets were cultured overnight in RPMI-1640 medium containing 11 mM glucose, 10% FBS, and 2 mM glutamine. After overnight recovery, islets were incubated in KRB buffer with 2.8 mM glucose and 0.5% BSA for 60 min, transferred to a 96-well plate with 4 islets per well containing 200 μl/well of compound solutions prepared in appropriate glucose concentrations and 0.1% BSA, and incubated at 37°C for 60 minutes. Secretion was stopped by refrigerating the plates at 4°C for 3 minutes. Supernatant was removed from the wells and assayed for insulin levels using the MA6000 Mouse/Rat Insulin Kit (Meso Scale Discovery). For inhibitor treatment experiments, after 24 hours recovery, islets were cultured either overnight in the presence of PTX (100 ng/mL) or 1 hour in the presence of UBO-QIC (0.3 μM) before insulin secretion assay was performed.

### Data analysis

Dose response data were analyzed by four-parameter curve fit in Graphpad Prism 6. When applicable, maximal efficacy of each treatment condition was normalized to the maximal signal obtained in the same cell line from L-Trp without any G protein inhibitor as 100%, and reported as relative Emax in tables. Minimum significant ratio (MSR) of EC_50_ and minimum significant difference (MSD) of Emax in IP-1, cAMP, and phospho-ERK assays determined as previously described [6] were approximately 3-fold and 20%, respectively. Therefore, >3-fold differences in EC_50_ and >20% differences in Emax were considered significant. For group-wise comparisons, one-way ANOVA with Dunnett’s post-hoc test was used, and P values less than 0.05 were considered significant.

## Results

### GPR142 agonists induce both Gq and Gi-coupled signaling

**Fig 1:**
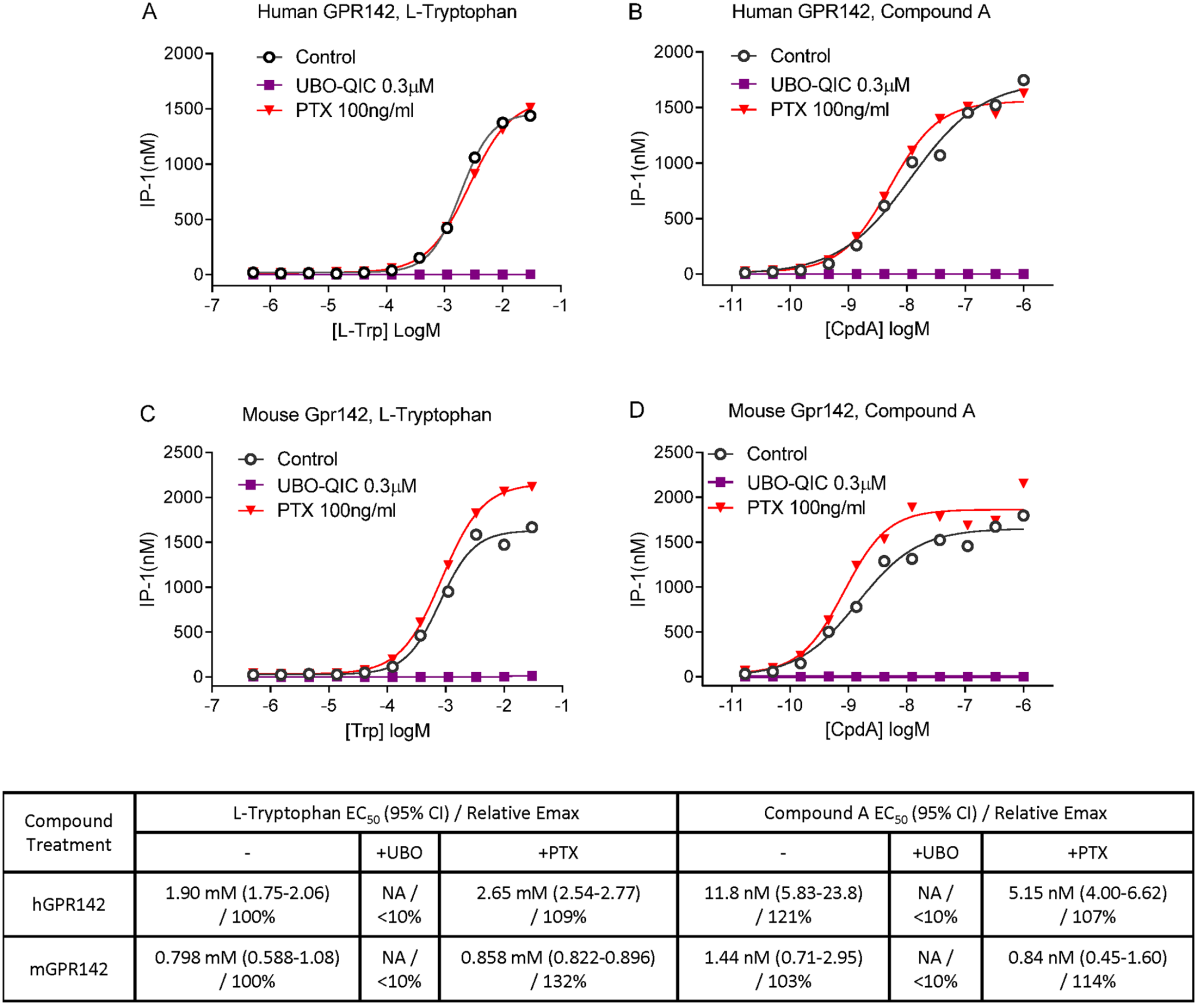
IP-1 accumulation induced by GPR142 agonists is Gq-dependent and Gi-independent. IP-1 levels in HEK293 cells expressing (A-B) human GPR142 or (C-D) mouse GPR142 treated with varying concentrations of (A, C) L-Trp or (B, D) Compound A, with or without different G protein inhibitors. Representative data of 3 independent experiments are shown. EC_50_ values, 95% confidence interval of EC_50_, and relative Emax are reported. NA: EC_50_ cannot be determined.

**Fig 2:**
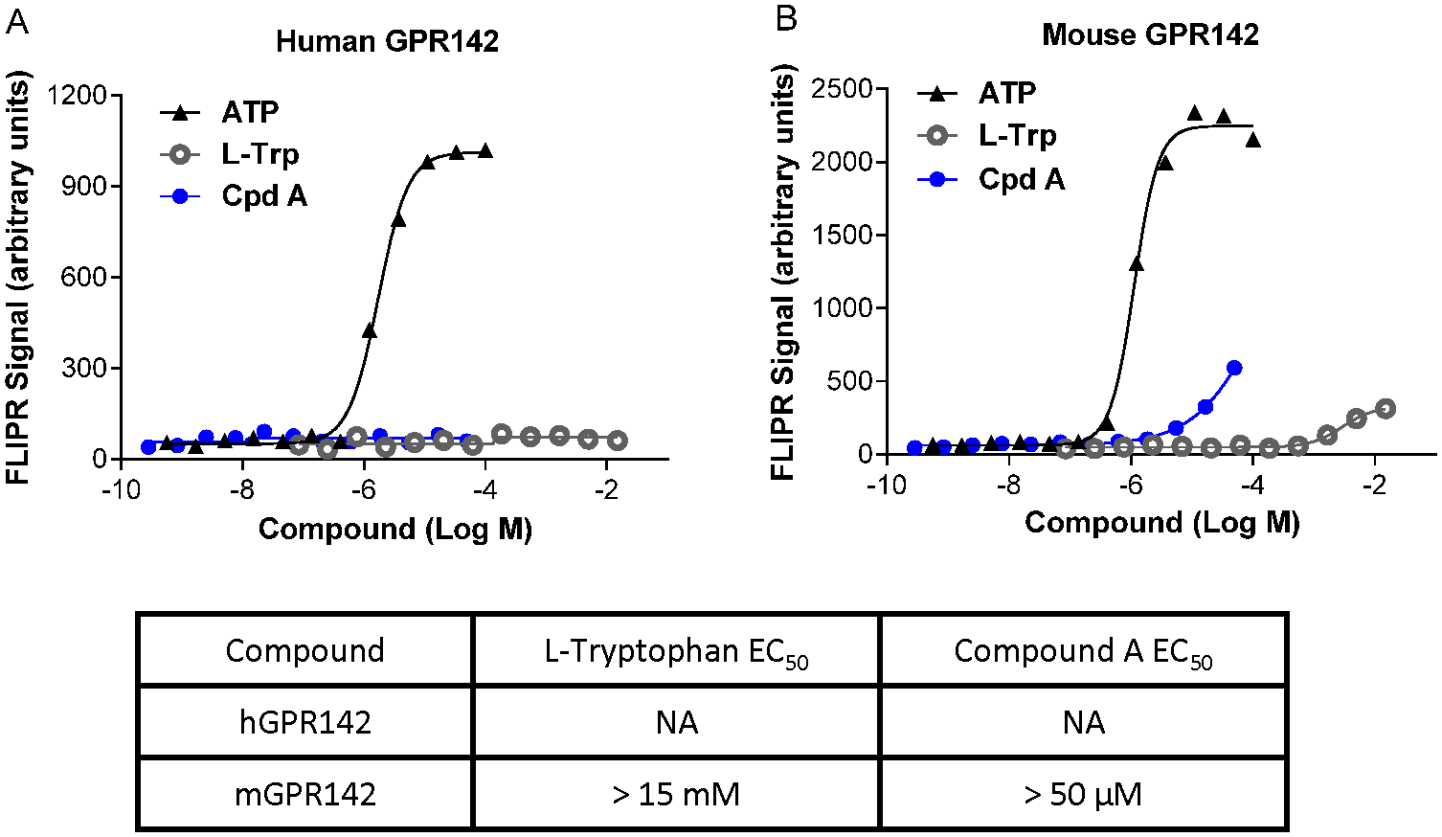
GPR142 agonists do not induce acute calcium flux. Calcium flux quantified by FLIPR in HEK293 cells expressing (A) human GPR142 or (B) mouse GPR142 treated with varying concentrations of ATP, L-Trp, or Compound A. Representative data of 3 independent experiments are shown.

**Fig 3:**
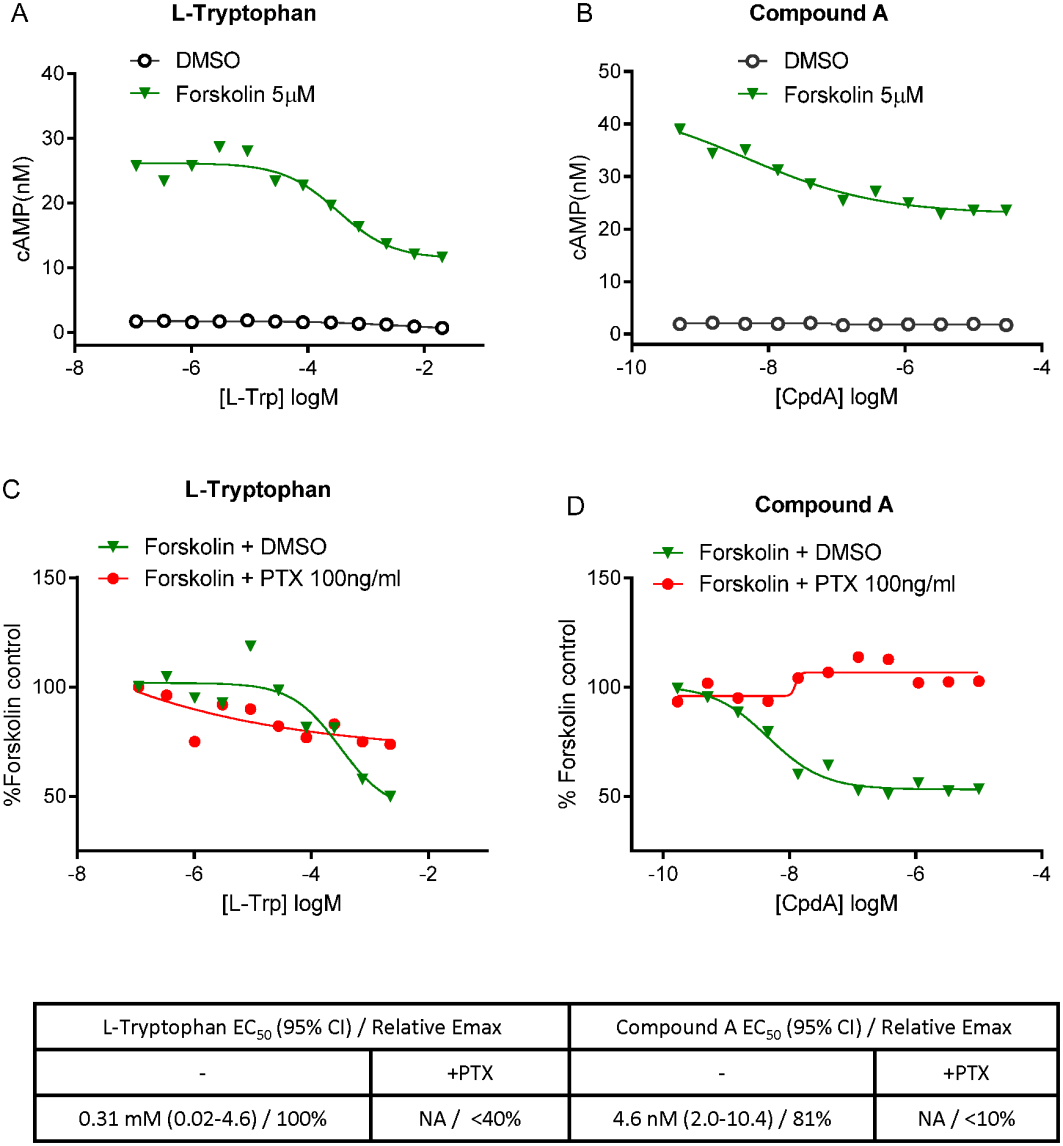
GPR142 agonists suppress cAMP via Gi signaling. (A-B) cAMP levels in HEK293 cells expressing human GPR142 treated with or without 5μM forskolin and with varying concentrations of L-Trp (A) or CpdA (B). (C-D) cAMP levels in forskolin-stimulated HEK293 cells expressing human GPR142, either pretreated with 100 ng/ml PTX or DMSO control, and treated with varying concentrations of L-Trp (C) or CpdA (D). Data are expressed as percentage normalized to cells treated with 5μM forskolin and no addition of GPR142 agonist compounds or PTX. Representative data of 3 independent experiments are shown. EC_50_ values, 95% confidence interval of EC_50_, and relative Emax are reported. NA: EC_50_ value cannot be determined.

Activation of GPR142 was previously reported to lead to accumulation of myo-inositol phosphate [7], suggestive of Gq coupling. However, the requirement for Gq signaling in the function of the GPR142 transcript isoform expressed in pancreatic islets has not been definitively demonstrated by specific inhibitors of Gq function. Furthermore, whether GPR142 activation triggers intracellular calcium mobilization, a prototypic response of Gq signaling, is also unknown. To examine G protein-coupled signaling downstream of GPR142, we generated HEK293 cell lines overexpressing either human or mouse GPR142 and measured intracellular levels of D-myo-inositol 1-phosphate (IP-1), a downstream metabolite of D-myo-inositol 1,4,5-triphosphate (IP-3), in these cells. Both L-Tryptophan (L-Trp), a natural ligand for GPR142, and a synthetic and selective GPR142 agonist compound A (N-[(3-methylimidazol-4-yl)methyl]-1-[5-methyl-4-(2-thienyl)pyrimidin-2-yl]-5-propyl-pyrazole-4-carboxamide) [8](example 49), were able to trigger IP-1 accumulation after compound treatment for one hour in cells with heterologous expression of GPR142 but not in untransfected control HEK293 cells (Fig 1). Intriguingly, we were not able to detect significant intracellular calcium flux during acute GPR142 agonist treatment (≤ 2 minutes) using the Fluorescent Imaging Plate Reader (FLIPR) technology even at concentrations several orders of magnitude higher than what was sufficient to induce IP-1. Importantly, in the same cellular system the positive control adenosine triphosphate (ATP) elicited dose-dependent calcium flux (Fig 2)[9]. To assess potential involvement of other Gα subunits, including Gs and Gi, we measured intracellular cyclic AMP (cAMP) levels. L-Trp or CpdA did not induce cAMP accumulation in GPR142-expressing cells in the absence of the adenylyl cyclase activator forskolin (data not shown), while in the presence of forskolin, GPR142 activation by L-Trp or CpdA suppressed cAMP accumulation (Fig 3). These data suggest that GPR142 activate Gi-coupled signaling but doesn’t signal through Gs. We used specific inhibitors for Gq and Gi signaling to further examine pathways downstream of GPR142 activation. Addition of UBO-QIC (also known as FR900359), a cyclic depsipeptide isolated from *Ardisia crenata* that specifically inhibits Gq/11-coupled signaling events [10], completely abolished IP-1 accumulation caused by L-Trp or CpdA in GPR142-expressing cells, while pretreatment of Pertussis toxin (PTX), a protein-based AB5-type exotoxin that catalyzes the ADP-ribosylation and inactivation of Gi subunits, did not significantly affect IP-1 levels (Fig 1). Conversely, pretreatment with PTX blocked the ability of GPR142 agonists to suppress cAMP (Fig 3). These data demonstrate that GPR142 agonists indeed activate both Gq and Gi signaling.

### ERK phosphorylation and dynamic mass redistribution (DMR) upon GPR142 activation exhibits differential requirements for Gi and Gq-coupled signaling

**Fig 4:**
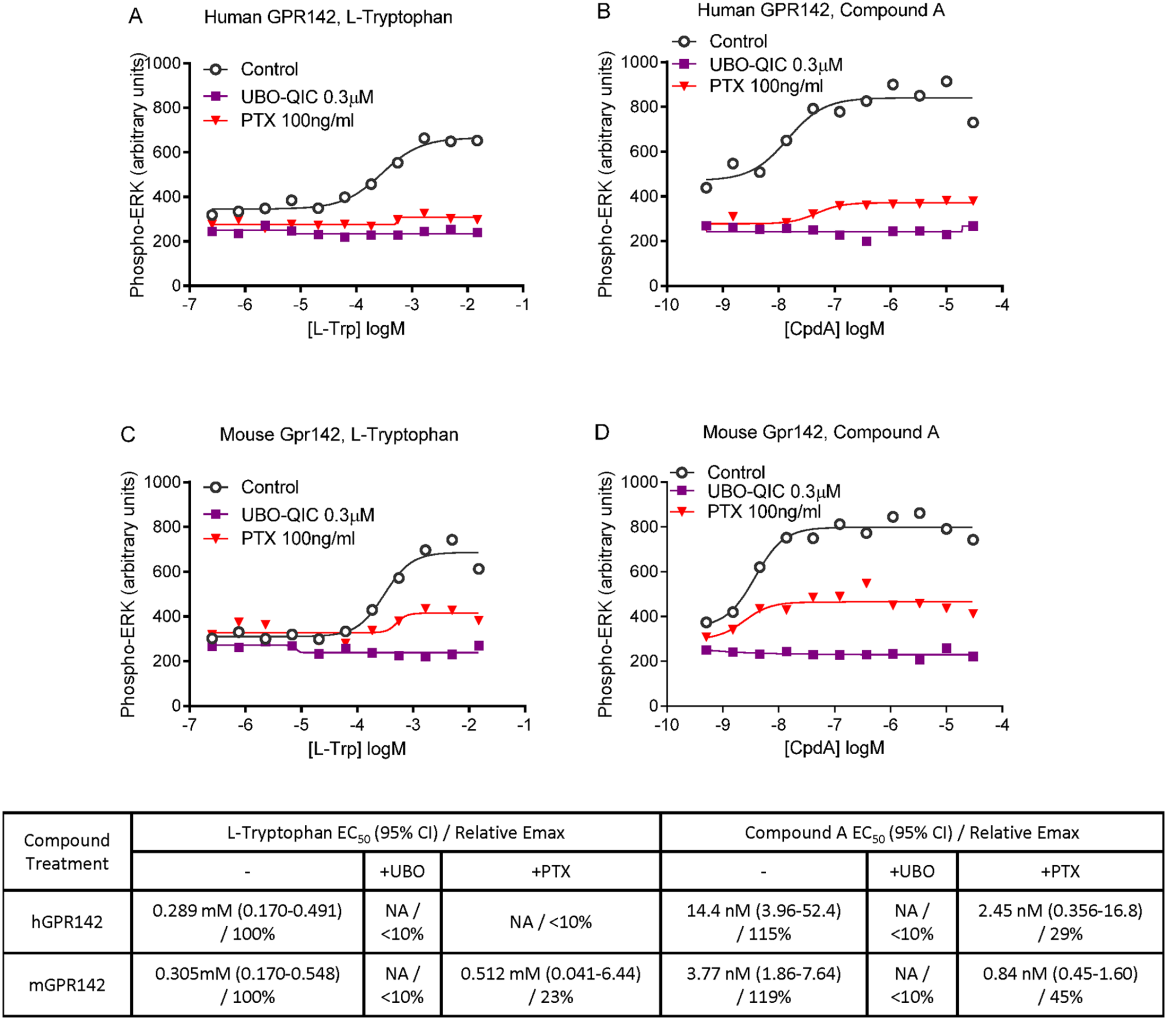
ERK phosphorylation upon GPR142 activation requires both Gq and Gi-coupled signaling. Levels of phospho-Thr202/Tyr204 ERK in HEK293 cells expressing (A-B) human GPR142 or (C-D) mouse Gpr142 treated with or without different G protein inhibitors and with varying concentrations of L-Trp (A, C) and CpdA (B, D). Representative data of 3 independent experiments are shown. EC_50_ values, 95% confidence interval of EC_50_, and relative Emax are reported. NA: EC_50_ value cannot be determined.

**Fig 5:**
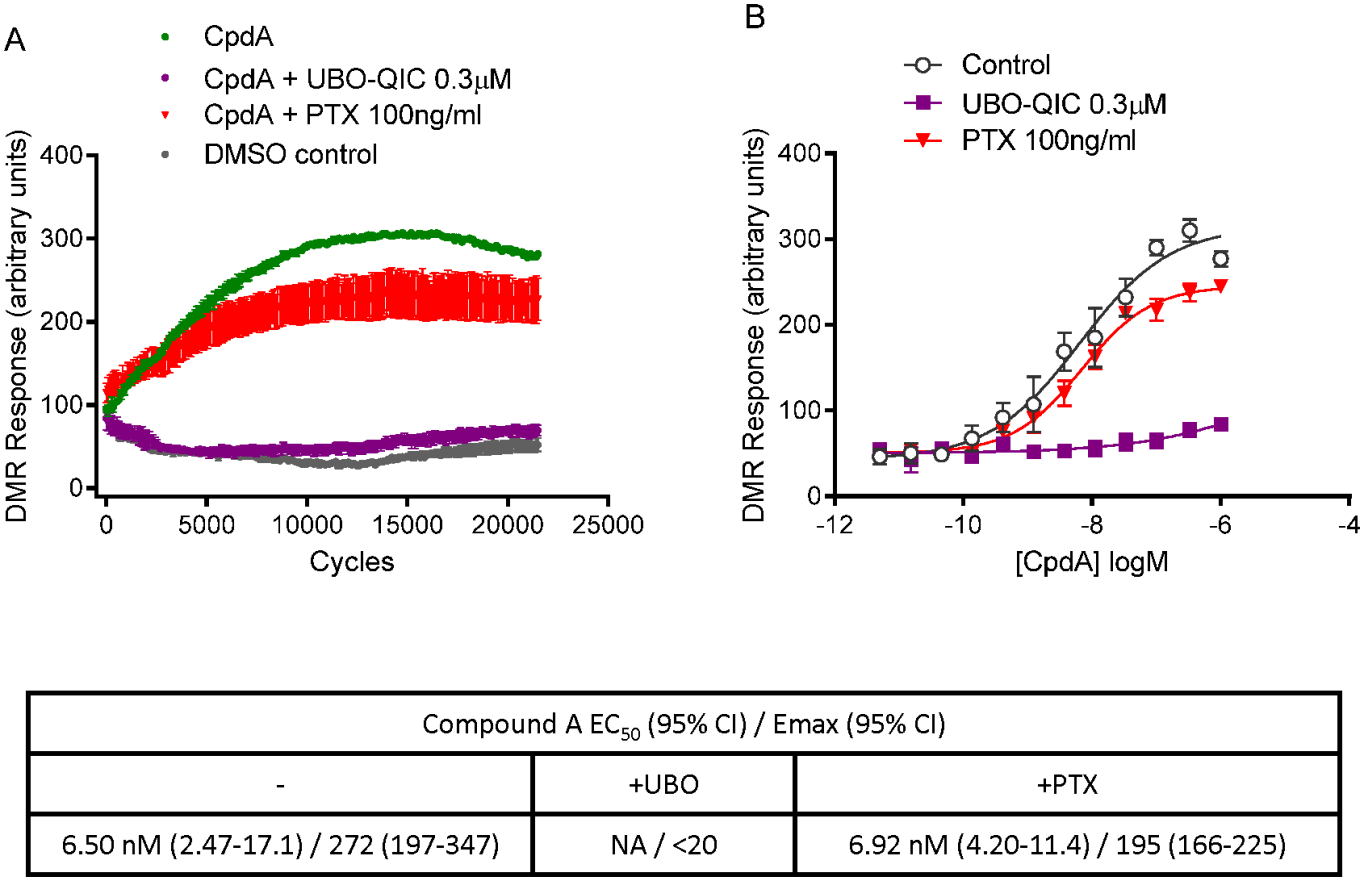
DMR signal upon activation of GPR142 is Gq-dependent and largely Gi-independent. (A) Traces of label-free DMR assays in HEK293 cells expressing human GPR142 incubated in the presence of 0.1μM CpdA and treated with or without G protein inhibitors. Representative data of 3 independent experiments are shown. (B) Concentration response curve of CpdA in HEK293 cells expressing human GPR142 treated with or without G protein inhibitors. The maximal DMR signal reached within 20000 cycles at each concentration was used to determine the concentration response curve. Data are mean ± SEM of four replicate wells. Representative data of 3 independent experiments are shown. EC_50_ values, 95% confidence interval of EC_50_, Emax (in arbitrary units), and 95% confidence interval of Emax are reported.

We examined whether GPR142 activation can induce ERK phosphorylation, which is a downstream GPCR signaling mediator that can be triggered by both G protein-dependent and independent pathways [11]. We found that L-Trp and CpdA could induce ERK phosphorylation in a dose-dependent manner in HEK293-GPR142 cells but not in control HEK293 cells (Fig 4 and data not shown). UBO-QIC completely and PTX partially inhibited phosphorylation of ERK induced by GPR142 agonists, consistent with both Gq- and Gi-coupled signaling downstream of GPR142 leading to ERK phosphorylation. DMR is a label-free whole-cell phenotypic assay that can be used to monitor functional consequences of GPCR pharmacology in living cells in real-time [12]. We found that GPR142 agonist CpdA triggered dose-dependent DMR responses in HEK293-GPR142 cells but not in control HEK293 cells, and this activity is completely abolished by UBO-QIC (Fig 5). In contrast, PTX pretreatment did not affect the potency of CpdA in the DMR assay, though it led to a non-significant decrease in the maximal DMR signal. These data indicate the DMR signal triggered by GPR142 activation is Gq-dependent, while Gi plays a minimal role in this response.

### Stimulation of insulin secretion by GPR142 agonists in pancreatic islets requires Gq-coupled signaling and is glucose-dependent

**Fig 6:**
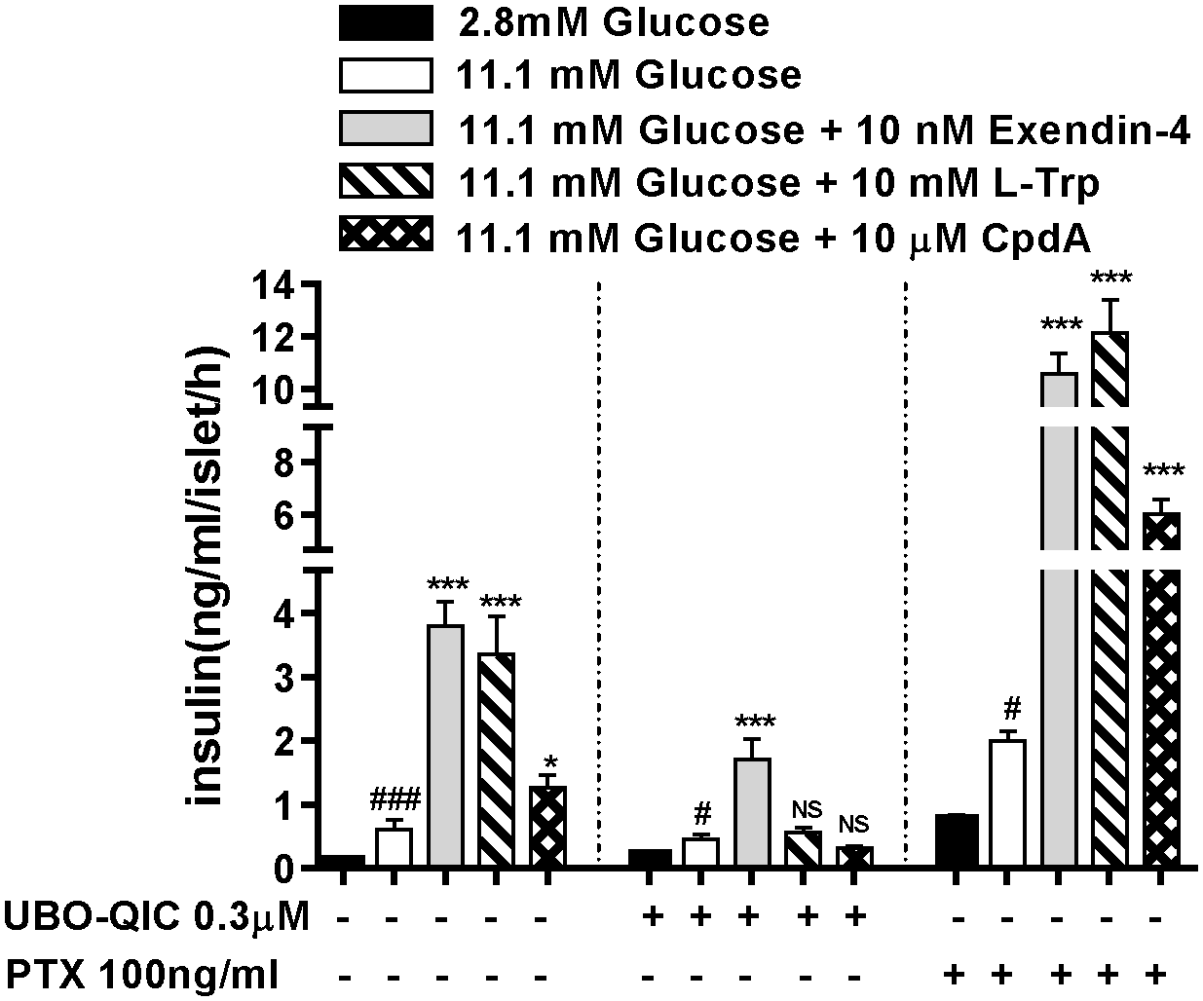
Gq-coupled signaling mediates GPR142 activation induced GSIS in islets. Insulin secretion was examined in pancreatic islets isolated from normal male C57 mice. GPR142 agonist triggered insulin secretion was assayed by incubating 4 islets per well in Kreb’s buffer in absence or presence of different G protein inhibitors. Data are shown as mean ± s.e.m (n = 6 wells per treatment). *p<0.05, ***p<0.001 for GPR142 agonist stimulated islets compared to 11.1mM glucose group. NS: not significantly different for GPR142 agonist stimulation compared to 11.1mM glucose group. #p<0.05, ###p<0.001 for 11.1mM glucose group compared to low glucose group.

**Fig 7:**
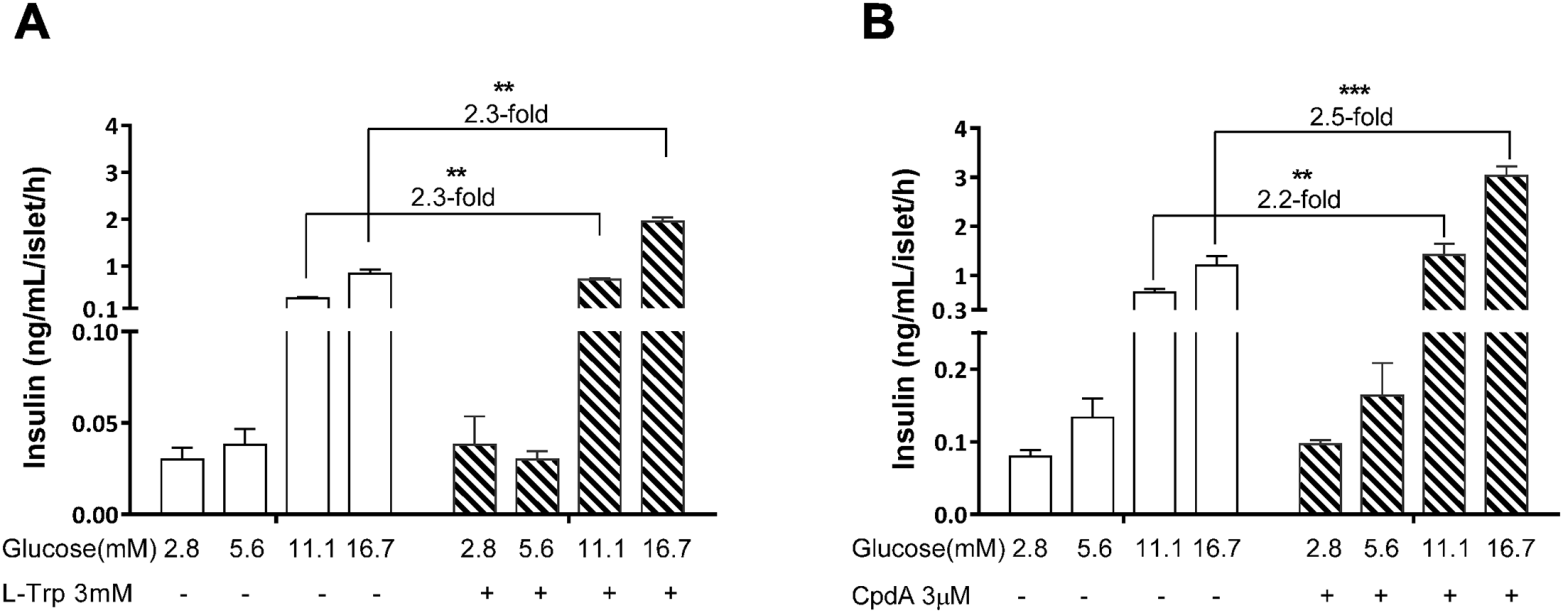
Stimulation of insulin secretion by GPR142 agonists is glucose-dependent. Insulin secretion was examined in pancreatic islets isolated from normal male C57 mice. Islets were incubated in the presence of GPR142 agonists L-Trp (A) or CpdA (B) at various ambient glucose concentrations. Data are shown as mean ± SEM (n = 6). **p<0.01, ***p<0.001 for GPR142 agonist stimulated islets compared to the vehicle group at the same glucose concentration.

We examined the requirement for different Gα subunits in GPR142 mediated insulin secretion using pharmacological inhibitors for Gq or Gi signaling. The Gq inhibitor UBO-QIC completely abolished the stimulatory effects on insulin secretion of L-Trp and CpdA in primary murine pancreatic islets (Fig 6). UBO-QIC treatment also had a mild inhibitory effect on high glucose induced insulin secretion, consistent with a report of impaired glucose-induced β cell depolarization in G_q/11_-deficient mice [13]. Conversely, the Gi inhibitor PTX increased baseline insulin release as expected, but did not impair the stimulatory effects of GPR142 agonists on insulin secretion (Fig 6). Neither UBO-QIC nor PTX significantly affected the ability of Exendin-4, a GLP-1 receptor agonist that activates Gs signaling, to enhance insulin secretion. We further examined the effect of GPR142 agonists on insulin secretion at various ambient glucose concentrations. Both L-Trp and CpdA enhanced insulin secretion only in the presence of high glucose but not under low glucose conditions (Fig 7). These data indicate stimulation of insulin secretion by GPR142 agonists requires Gq-coupled signaling and is strictly glucose-dependent.

## Discussion

In the present study, we surveyed cellular responses triggered by natural and synthetic GPR142 agonists L-Trp and CpdA in cells expressing human or mouse GPR142. Utilizing a Gq-specific inhibitor UBO-QIC, we provide definitive evidence that Gq signaling downstream GPR142 activation is responsible for IP accumulation, leads to ERK phosphorylation, mediates whole-cell changes manifested as a robust DMR signal, and is critically required for potentiation of glucose-dependent insulin secretion in pancreatic islets. In addition, we describe a novel finding that GPR142 agonists stimulate Gi signaling when the receptor is recombinantly expressed in HEK293 cells, which significantly contributes to ERK phosphorylation, but does not significantly impact whole-cell responses detected by DMR or affect GPR142’s insulin secretagogue activity in pancreatic islets.

**Fig 8:**
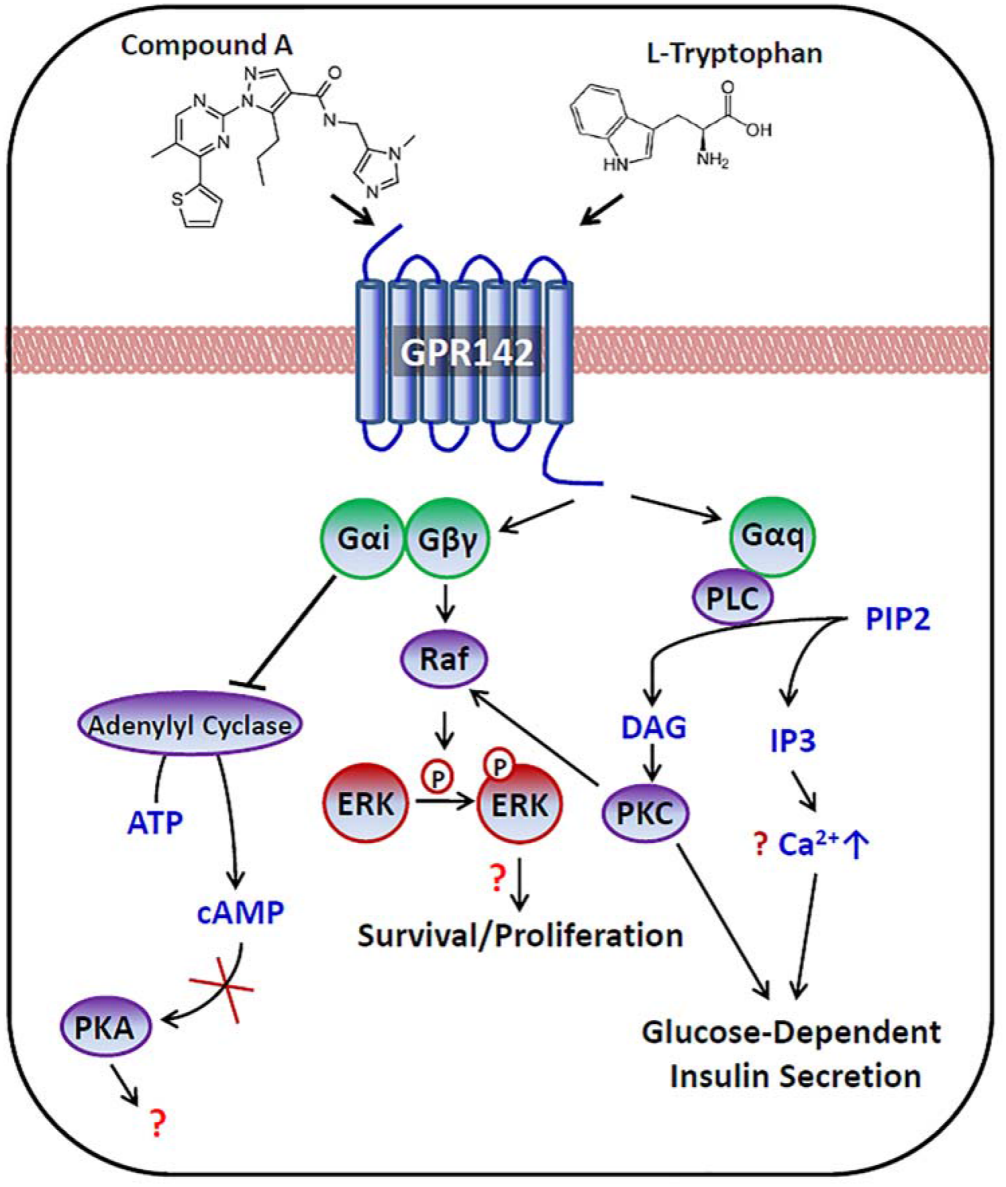
Proposed model of GPR142 mediated signaling pathways in β cells. Stimulation of GPR142 triggers activation of Gq signaling and IP-3 formation, which is critical for enhancement of glucose-dependent insulin secretion. Activation of GPR142 can also result in the activation of Gi and suppression of cAMP, the contribution of which to GPR142 function remains to be determined. Activation of both Gq and Gi signaling by GPR142 agonism result in the phosphorylation of ERK, which may contribute to the previously reported beneficial effects on β cell survival and/or proliferation for GPR142 agonists [17].

Activation of Gq subunits promotes phospholipase C (PLC)-mediated generation of second messengers diacylglycerol (DAG) and IP-3. DAG stimulates the activity of different isoforms of protein kinase C (PKC), while IP-3 triggers the release of calcium from endoplasmic reticulum stores (Fig 8). These signaling events in β cells upon activation of other GPCRs have been shown to play key roles in promoting insulin secretion in a glucose-dependent manner [14]. Our findings that the insulin secretagogue activity of GPR142 agonists is only evident under high ambient glucose conditions and can be fully blocked by a Gq inhibitor are consistent with Gq coupling of the receptor. It is intriguing that GPR142 agonists induced robust IP accumulation but had minimal effect on calcium flux in the HEK293 cell system we used. It’s possible that the kinetics of receptor signaling led to the different activities in these two assays, since we could only examine acute calcium fluxes with FLIPR technology, while the IP signal is allowed to accumulate over time after GPR142 agonist treatment. Alternatively, it’s possible that calcium flux downstream of GPR142 activation integrates both Gq signaling and outputs of other pathways, leading to a net result of no overt increase in calcium in the cellular system we examined. There’s precedence in the literature that Gi-coupled GPCRs could inhibit calcium currents through either Gα or Gβγ-dependent mechanisms [15, 16]. Further mechanistic exploration will be needed to better elucidate the signaling mechanisms of this receptor.

The novel finding that GPR142 can also trigger Gi signaling is supported by the observation that, in the HEK293 cell system, GPR142 agonists suppress forskolin-induced cAMP accumulation, which in turn can be abrogated by the Gi inhibitor PTX. Further, PTX partially attenuates GPR142 agonists’ effects on ERK phosphorylation. However, whether Gi-coupled signaling plays a physiological role in GPR142 function in native cell types with endogenous expression of this receptor is unknown. In recombinant cell systems, the relative stoichiometry of receptor to G proteins may be different from that in native cells. Under conditions where the receptor is in excess, e.g. when overexpressed in a recombinant cell line, the formation of ternary complexes of the agonist-occupied receptor with additional heterotrimeric G proteins may be favored. In fact, it has been previously reported for various GPCRs that increases in receptor density can lead to cellular responses not found under conditions of lower receptor expression [18]. In addition, the relative expression levels of various α, β, and γ subunits are cell type-dependent, resulting in different repertoires of heterotrimeric G proteins in different cell types. This may also have a significant impact on the formation of ternary complexes upon agonist-receptor binding. Indeed, 8 different α subunits of the Gi/Go class, as well as 5 β subunits and 12 γ subunits have been described, altogether comprising a large number of possible heterotrimeric G proteins of the Gi/Go family [19].

It’s also worth noting that recently reported cell type-specific RNAseq data from pancreatic islets suggest GPR142 mRNA is expressed in both β cells and non-β endocrine cells [20]. Thus, it’s formally possible that GPR142 activation could trigger Gi signaling in non-β cells, such as the Somatostatin-producing δ cells, in turn having an indirect effect on β cell insulin secretion via intra-islet paracrine signaling. However, our data show the Gi inhibitor PTX does not affect the ability of GPR142 agonists to stimulate insulin secretion, clearly indicating that Gq signaling activity plays a predominant role in GPR142-mediated stimulation of insulin secretion in primary mouse islets. In addition to pancreatic islets, GPR142 transcript is also enriched in murine gastric ghrelin cells [21]. Interestingly, several other nutrient-sensing GPCRs that couple to both Gq and Gi signaling, including Free fatty acid receptor 2 (FFAR2/GPR43) and Free fatty acid receptor 4 (FFAR4/GPR120) were reported to preferentially couple to Gi in gastric ghrelin cells and suppress ghrelin secretion, potentially due to a high enrichment of several Gi/o subunits in this cell type [21]. Whether the function of GPR142 in gastric ghrelin cells is mediated by Gi signaling remains to be determined, although a recent report showing L-Trp stimulating, rather than inhibiting, ghrelin secretion [22] suggests GPR142-mediated Gi signaling may not account for this activity.

ERK phosphorylation has been linked to cell survival and proliferation in multiple cell types, including pancreatic β cells [23, 24]. The receptor for the incretin hormone glucose-dependent insulinotropic polypeptide (GIP) was recently reported to promote β cell survival via phospho-ERK-dependent activation of Tcf1 [25]. Our report is the first to demonstrate that GPR142 agonists induce ERK phosphorylation via both Gq- and Gi-dependent mechanisms. As GPR142 agonists have also been linked to potential beneficial effects on β cell health, including improved survival and increased proliferation in vitro [17], it will be interesting to determine the role of the ERK phosphorylation, Tcf1, as well as contributions of Gq and Gi signaling to these effects.

In summary, our data indicate GPR142 agonism as a novel therapeutic approach for the treatment diabetes has minimal risk for hypoglycemia. Moreover, cellular assays reflecting Gq signaling events as well as the whole-cell DMR assay may be used for high-throughput screening approaches. Further investigation of additional second messengers upon GPR142 activation may be warranted to delineate the signaling events responsible for the receptor’s additional physiological functions and pharmacological potential.
